# Comparison of a novel bovine respiratory disease prediction technology and an automated animal disease detection technology to traditional methods in a U.S. feedlot

**DOI:** 10.1093/tas/txaf067

**Published:** 2025-05-18

**Authors:** Brian S Schupbach, Michael S Davis, Tracy D Jennings, Andrea L Dixon, David G Renter, Jason S Nickell

**Affiliations:** Merck Animal Health, Lenexa, KS 66219USA; Bos Technica Research Services, Salina, KS 67401USA; Merck Animal Health, Lenexa, KS 66219USA; Center of Outcomes Research and Epidemiology, College of Veterinary Medicine, Kansas State University, Manhattan, KS 66506USA; Center of Outcomes Research and Epidemiology, College of Veterinary Medicine, Kansas State University, Manhattan, KS 66506USA; Merck Animal Health, Lenexa, KS 66219USA

**Keywords:** antimicrobial, bovine respiratory disease, cattle, disease detection, feedlot

## Abstract

The objectives of this study were to evaluate feedlot cattle health and performance among three different bovine respiratory disease (BRD) control programs and two different disease detection modalities (i.e., a 3 × 2 factorial design). The BRD control treatments consisted of 1) Negative control, 2) Positive control (Tildipirosin to 100% of the group), and 3) Targeted BRD control program (TBCP) based on individualized risk prediction generated by a novel technology ([Whisper On Arrival; Merck Animal Health] ± Tildipirosin based on a proprietary algorithm). The disease detection treatments consisted of 1) cattle monitored exclusively by a novel animal disease detection (ADD) technology (SenseHub Feedlot; Merck & Co., Inc., Rahway, NJ, USA and its affiliates), or 2) cattle monitored by traditional pen-riding (PR) methods. Auction market-derived beef calves were procured by traditional means, transported to a single site, and randomly allocated to one of six treatment groups within each block. The study population was followed to closeout (224 d). Data were analyzed as a completely randomized block design within a 3 × 2 factorial treatment format. No interactions (*P* values > 0.05) between BRD control practices or disease detection methods were observed in this study. Across the BRD control treatments, the TBCP reduced BRD control antimicrobial use by 25% compared to the positive control. However, the positive control displayed improvement (*P* values ≤ 0.05) in BRD morbidity, overall removals, and overall mortality at the time of closeout compared to the negative control and the TBCP. Regarding disease detection, compared to cattle monitored by PR methods, cattle monitored by the ADD technology displayed a reduction (*P* values ≤ 0.05) in days to first BRD treatment, pen-deads, and overall removals. Cattle monitored by ADD technology displayed an increase (*P *= 0.06) in net financial value of $29.50/head compared to cattle monitored by PR methods.

## Teaser

Within this clinical trial, a traditional bovine respiratory disease (BRD) control program was found to be superior in health, performance, and carcass metrics relative to a negative control and a targeted BRD control program. Likewise, a novel automated disease detection system was observed to reduce the negative health impacts of BRD compared to traditional pen riding methods.

## INTRODUCTION

Although various disease syndromes (infectious and noninfectious) impact post-weaned beef cattle, it is understood that bovine respiratory disease (BRD) is the predominant disease condition observed in weaned cattle reared in backgrounding, stocker, and feedlot production systems (Griffin, 1997; Brooks and Ward, 2011; USDA, 2011). The biology of the BRD complex is multifactorial and reflects the epidemiological triad whereby the disease is manifested by interactions among the animal, the pathogen(s), and the animal’s respective environment (Apley, 2006; Taylor et al., 2010). Therefore, management of the BRD complex must also be multifactorial and focus on the animal (and its associated cohort), the array of pathogens, and the environment.

The inability of stockmen to provide timely and accurate therapy to animals stricken with BRD may increase the likelihood of lung pathology, negative feed performance, and mortality. Among cattle surviving the initial disease insult, presence of chronic lung pathology at the time of harvest has previously displayed a significant reduction in average daily gain (ADG) compared to animals without lung lesions (Wittum et al., 1996; Bryant et al, 1999; Gardner et al., 1999; Thompson et al., 2006; Reinhardt et al., 2009; Rezac et al., 2014). Additionally, cattle with evidence of lung pathology at harvest reflect both cattle with and without history of BRD treatment, potentially reinforcing diagnostic challenges (Wittum et al., 1996; Gardner et al., 1999; Thompson et al., 2006).

Multiple management tools are commonly executed to minimize the negative attributes of BRD in post-weaning production systems (Galyean et al., 2022). Antimicrobial BRD control programs (i.e., metaphylaxis) have repeatedly displayed efficacy in reducing the negative impacts of BRD (Kilgore et al., 2005; Rooney et al., 2005; Nickell et al., 2008; Nickell and White, 2010; Ives and Richeson, 2015). Metaphylaxis is applied to a population of cattle who, based on history of exposure to known BRD risk factors, are at various stages of BRD disease status. This practice has previously displayed tremendous financial value by improving cattle health and performance (Dennis E.J et al., 2018). However, consumer perception regarding the practice of antimicrobial use in food animal production places scrutiny on this management practice (Manyi-Loh et al., 2018; Barrett et al., 2021). A targeted BRD control program (TBCP) based on individualized risk prediction generated by a novel technology (Whisper On Arrival; Merck Animal Health, Rahway, NJ) has been previously described (Nickell et al., 2021). Prior research has reported this technology can reduce BRD control antimicrobial use by up to 43% without a loss in health, performance, and carcass outcomes compared to conventional metaphylaxis practices (Nickell et al., 2021). However, in that study population there was relatively low disease pressure as evidenced by the low mortality outcomes among calves in the negative control treatment (Nickell et al., 2021). Therefore, assessment of the technology in a population destined to be at a higher risk of developing BRD is necessary to assess the product’s robustness in its current state.

Although a standardized BRD diagnosis does not currently exist, a commonly applied BRD diagnostic modality reflects the initial detection of BRD clinical signs via human observation followed by a rectal temperature that meets and/or exceeds a predetermined threshold (Booker et al., 1997; Perino and Apley, 1998; Jim et al., 1999; Schunicht et al., 2007; Perrett et al., 2008; Hannon et al., 2009). Although widely applied, this current diagnostic application has previously shown to be inaccurate (White and Renter, 2009; Theurer et al., 2014; Timsit et al., 2016). Consequently, the inability to accurately diagnose BRD may have a detrimental impact on economic viability, animal welfare, and judicious antimicrobial use. Additionally, a shrinking labor force places added pressure on an already inaccurate system thereby necessitating an improvement in accuracy and efficiency of animal handlers in these previously stated production systems (Zahniser et al., 2018). The automated disease detection (ADD) technology (SenseHub Feedlot; Merck & Co., Inc., Rahway, NJ, USA and its affiliates) is a novel health detection system implementing artificial intelligence methods to simultaneously analyze body temperature and activity in a continuous manner throughout the feeding period. The system consists of a small hardware footprint which includes a wearable device (a biometric recording ear tag applied in the left ear) and a strategically placed antenna. The system utilizes a long-range wide-area (LoRa) operating system thereby covering relatively large distances with minimal hardware. This ADD technology is not specific for BRD; rather, the system is designed to identify outliers (i.e., individual animals) in the respective population. An automatically generated list of animals identified as outliers (in their respective pen) is delivered to the user every morning via email. Tags on the alerted animals subsequently illuminate and rapidly blink for several hours to assist in the identification of cattle in their respective pen(s).

It is noteworthy to emphasize that disease detection does not equate to disease diagnosis. Disease detection refers to the screening of a specific population that may or may not yet be exhibiting clinical signs of disease (Dohoo et al., 2003). The goal of a screening test is to detect the syndrome of interest before the condition becomes clinically apparent. However, a final diagnosis is determined by a subsequent confirmatory test (Dohoo and Stryhn, 2003). Ultimately, the intention is to elevate the probability of a true diagnosis. For example, the traditional BRD diagnosis utilizes human observation of outward clinical signs (e.g., depression, nasal discharge) to *detect* disease while the follow-up rectal temperature data has traditionally *confirmed* apparent disease status (Perino and Apley, 1998). In the current ADD system, once the alerted animal (i.e., the outlier) has been segregated, further examination by trained personnel is then necessary to confirm disease status.

The primary objectives of this study were to 1) compare health, performance, and carcass metrics among high risk beef-breed feedlot cattle whose BRD control decision was based on the TBCP technology to a positive control (PC; conventional BRD control practice where 100% of the population receives BRD control therapy) and a negative control (NC), and 2) compare health, performance, and carcass metrics among cattle monitored by ADD directly to conventional pen riding (PR) practices utilizing a traditional BRD diagnosis.

The secondary objectives of this study were to 1) evaluate the interaction of said BRD control and disease detection practices, and 2) estimate economic outcomes specifically for the ADD technology relative to the PR modality. The results of this study are expected to be relevant to both backgrounder and feedlot production systems (i.e., confined cattle systems managing animals in an all-in/all-out method) by means of data collected at both 60 d on feed and closeout, respectively.

## MATERIALS AND METHODS

Institutional Animal Care and Use Committee (IACUC) approval was not obtained by the study site. However, guidelines stated in the Guide for the Care and Use of Agricultural Animals in Agricultural Research and Teaching (FASS, 2010) were followed. The study was constructed as a clinical trial in a 3 × 2 factorial treatment format (**Table 1**) within a completely randomized block design. The study was carried out at one site in Oklahoma, sponsored by Merck Animal Health, and executed by Bos Technica Research Services, Inc.

**Table 1.** Overview of the current study design. This study was designed as a 3 × 2 factorial study where the main effects included three bovine respiratory disease (BRD) control strategies and two disease detection strategies. The study population was composed of auction-market derived beef/beef-cross heifers in one Oklahoma feedlot

		Disease detection program^2^		
		ADD	PR	
** BRD control program** ^1^	NC	Treatment 1 (6 pens)	Treatment 2 (6 pens)	12 pens
	PC	Treatment 3 (6 pens)	Treatment 4 (6 pens)	12 pens
	TBCP	Treatment 5 (6 pens)	Treatment 6 (6 pens)	12 pens
		18 pens	18 pens	36 pens

^1^Three BRD control strategies were evaluated: Negative Control (NC), Positive Control (PC; 100% of treatment group received the BRD control antimicrobial which reflects traditional management), or a Targeted BRD Control Program (TBCP; individual animal prediction of BRD risk generated by a novel hardware/software system capturing cardiopulmonary data subsequently analyzed by machine learning techniques).

^2^Two disease detection methods were evaluated: an automated disease detection system (ADD; a novel hardware/software system designed to continuously capture animal temperature and activity and whose data is analyzed by machine learning techniques) and traditional pen riding (PR) practices which reflect conventional management composed of visual observation of clinical signs.

### Sample Population Description

The targeted sample population included weaned auction market-derived beef heifer calf lots considered to be “medium” to “high-risk” for development of BRD. The desired risk factors of this population reflected those previously reported as being associated with an increased risk of BRD development and sequelae (Cernicchiaro et al., 2012a, 2012b, c). Cattle were procured through standard industry channels. Upon arrival, only visually healthy calves were eligible for study enrollment. Calves displaying elevated clinical illness scores (≥ 2; **Table 2**) and/or signs of other non-BRD infectious or noninfectious syndromes were removed from the study population prior to allocation. Original calf lots remained together until the day of processing (Day 0).

**Table 2.** Clinical illness scoring system implemented in the present study. Clinical scores were utilized by study personnel to screen cattle at the time of enrollment and contributed to the case definition of cattle allocated to a traditional bovine respiratory disease detection method

Score	Description
0 = Healthy	Normal, healthy behavior
1 = Mild	May stand isolated with its head down or ears drooping; but will quickly respond to minimal stimulation
2 = Moderate	May remain recumbent or stand isolated with head down; may show signs of muscle weakness (standing cross-legged, knuckling, or swaying when walking), depression obvious when stimulated
3 = Severe	May be recumbent and reluctant to rise, or if standing isolated and reluctant to move; when moving, is ataxic, knuckling or swaying evident; head carried low with ears drooping; eyes dull, excess salivation/lacrimation possible, obviously gaunt
4 = Moribund	Unable to stand; approaching death; highly unlikely to respond to any antimicrobial treatment

Heifers were fed thrice daily using a single-axle, International truck equipped with a Harsh 575H mixer/delivery box. Load order for feed ingredients were chopped alfalfa hay, wheatlage (when applicable), sorghum silage (when applicable), steam flaked corn, choice white grease (when applicable), cane molasses (when applicable), supplement pellet, and micro ingredients. Upon addition of the last ingredient, all rations were mixed for 4 minutes at 1500 RPM.

Heifers were adjusted to a 94% concentrate diet using a series of four diets. Upon arrival, heifers were offered loose hay for the first 55 d on feed along with starter ration. After removal of loose hay, heifers remained on starter ration until study day 25. Heifers were fed Ration 2 for the next 9 d and then transitioned to Ration 3 until study day 41 when Ration 4 was fed. Monensin (Rumensin 90; Elanco US, Inc., Greenfield, IN) was included in all rations with concentration increasing from 25 g monensin/ton (DM basis) in Ration 1 to 44.4 g monensin/ton (DM basis) in Ration 4. Tylosin (Tylan 100; Elanco US, Inc., Greenfield, IN) was included in all rations at a level sufficient to provide 90 mg tylosin/heifer/d. Melengestrol acetate (MGA; Zoetis Animal Health, Parsippany, NJ) was supplied in finishing rations at a level of 0.5 mg/heifer/d. Heifers remained on Ration 4 until 36 to 29 d prior to harvest, at which time ractopamine (Optaflexx; Elanco US, Inc., Greenfield, IN) was added to the ration. Ractopamine was included in all feedings at a level of 27.3 g ractopamine/ton DM basis. Due to load size limitations, not all heifers in the trial could be fed using one load of feed per feeding. Consequently, two trucks were used to feed heifers in the trial, but loads were such that all heifers in a common statistical block were fed using the same truck.

Treatment diets were fed for ad libitum consumption and water was provided via a single continuous flow water trough located in pen. Feed amounts delivered to each pen were recorded manually by feed truck driver and electronically by feed truck scale system. A printout of the delivery amount was also produced at time of feeding.

Each statistical block reflected six pens (i.e., 3 × 2 factorial treatment design; **Table 1**) and only one block was enrolled on any given enrollment day. Once enough cattle were procured to meet the head count requirement for at least one study block, cattle were randomized to pens. Day 0 for the respective block (i.e., the day of randomization and processing) occurred within 96 hours post-arrival. Heifers within an arrival date and source were systematically gate-cut in groups of six and assigned to one of six pens until the desired head count for each pen within a block was attained. Heifers were not allowed access to feed or water during randomization to pens. The pen was then randomly assigned to one of six treatment groups (**Table 1**) using a random number generator www.microsoft.com. Immediately following randomization, heifers were weighed by pen on a 70 ft platform scale (Fairbanks TRBT88; Kansas City, MO) in one draft to determine starting weight.

On Day 0 (after randomization and pen weights had been obtained), each respective lot within a block was administered a processing regimen consisting of the following products: a modified-live viral vaccine (Vista Once SQ; Merck & Co., Inc., Rahway, NJ, USA and its affiliates), a multivalent clostridium toxoid (Vision 7; Merck & Co., Inc., Rahway, NJ, USA and its affiliates), a steroid implant (Revalor IH; Merck & Co., Inc., Rahway, NJ, USA and its affiliates), an oral deworming agent (Safe-Guard suspension 10%; Merck & Co., Inc., Rahway, NJ, USA and its affiliates), an injectable deworming agent (Dectomax; Zoetis Inc., Kalamazoo, MI), an abortifacient (Estrumate; Merck & Co., Inc., Rahway, NJ, USA and its affiliates), and (treatment group dependent; **Table 1**) an antimicrobial labeled for BRD control (Zuprevo; Merck & Co., Inc., Rahway, NJ, USA and its affiliates). Ear tissue was collected from each animal to test for persistent infection of Bovine Viral Diarrhea Virus (PI-BVDV). Each calf received both a visual dangle ear-tag with a unique visual identification number along with an individualized electronic identification (EID) tag. Finally, each calf within the ADD treatment group was administered the ADD biometric tag. The ADD biometric tag was paired with the calf’s respective visual identification and EID tag and applied to the left ear of the calf. On the day of collection, the PI-BVDV samples were subsequently transported to a commercial BVDV laboratory for testing purposes (Cattle Stats, LLC; Oklahoma City, OK). All BVDV results were received within 24 to 48 h of receipt at which time any positive animals were removed and quarantined from the study population.

It is accepted that a post-metaphylactic interval (PMI) be implemented after BRD control is administered to allow adequate time for the antimicrobial to generate its desired effect. In parallel, the ADD system requires a baseline of time to determine “normal” biometric outcomes to identify outliers. In this study, a two-day (Treatments 1 and 2; **Table 1**) or five-day (Treatments 3 to 6; **Table 1**) PMI was applied to the study population. After the respective PMI, calves were eligible for disease detection, subsequent diagnosis, and treatment (if applicable).

Unlike traditional BRD control and treatment studies where one group of individuals (i.e., pen riders) monitor the entire study population, one group of individuals was assigned to the ADD group (caretakers) while a separate group of individuals was assigned to the PR group (pen riders). Individuals were randomly assigned to be either pen rider or caretakers by means of a random number generator (Microsoft Office Excel 2007).

The BRD case definition differed between the two disease detection treatments. The BRD case definition of the PR group followed a traditional path which included a clinical illness score (CIS; **Table 2**) of 1 or 2 and a rectal temperature ≥ 40°C (Perino and Apley, 1998). Alternatively, a calf with a CIS of 3 (regardless of rectal temperature) also met the BRD case definition.

As the ADD system identifies outliers in the population of interest, the BRD case definition in the ADD group reflected cattle detected by the ADD software and displaying no clinical signs of non-BRD syndromes (e.g., bloat, neurological disease, lameness/injury) upon visual inspection and physical exam. A rectal temperature was captured among ADD monitored animals but was not part of the BRD case definition for this treatment group. Independently, ADD caretakers had the latitude to apply the PR BRD case definition (described above) to calves with presumptive BRD based on clinical signs (**Table 2**) if the calf was not alerted by the ADD system and was not within a post-treatment interval. However, ADD caretakers were instructed to only enter a pen if a calf had been alerted by the system or if a calf was observed with CIS ≥ 3 (**Table 2**). Across all treatments, calves observed with a CIS of 4 (**Table 2**) were subject to euthanasia at the discretion of feedlot personnel and/or the study investigator.

Once the PMI had expired, caretakers received an automated daily list from the ADD system. The list included only animals that were alerted by the ADD system and were not in a post-treatment interval (PTI; described below). These data included the pen number and visual identification number of each respective alerted animal. Subsequently, the light on the respective animal’s ADD tag would illuminate and rapidly blink for six hours to assist in locating the animal within the respective pen. All realistic attempts were made to mask caretakers and pen riders to observations of animals between the opposite disease detection treatments. For example, the daily lists generated by the ADD system were only available to the ADD caretakers; not to the individuals allocated to the PR group.

All calves in the study population were eligible for BRD treatment up to three times and the same BRD treatment regimen was applied across all treatments. The first BRD treatment consisted of florfenicol and flunixin meglumine (Resflor Gold; Merck & Co., Inc., Rahway, NJ, USA and its affiliates; 40 mg florfenicol/kg and 2.2 mg flunixin/kg; subcutaneous [SC] administration). Following a three-day PTI, cattle meeting the BRD case definition a second time received enrofloxacin (Baytril 100; Elanco Animal Health, Greenfield, IN; 12.5 mg/kg; SC). Following a three-day PTI, if the case definition was met a third time, affected calves were administered oxytetracycline (Biomycin 200; Boehringer Ingelheim Animal Health, Duluth, GA; 9 mg/kg; SC). After a final three-day PTI, if a calf met the respective case definition a fourth time, the calf was removed from the study population, a body weight was captured, and therapy was applied (at the discretion of study personnel), accordingly; however, the calf was considered off-study, and all data were collected up to the day of removal. At approximately 80 d on feed, all remaining calves within all lots were implanted with a commercial implant intended to last for the remaining duration of the feeding phase (Revalor 200; Merck & Co., Inc., Rahway, NJ, USA and its affiliates).

All calves that remained on study were followed to the point of harvest. Calves in each respective lot within a statistical block (6 pens) departed the feedlot on the same day and were harvested within the same shift. Individual carcasses were tracked by study personnel during the harvest process to ensure data integrity.

At the timepoints of reimplant and harvest, the ADD tags were assessed for placement, retention, and functionality. Successful tag retention was defined as being present in the ear and whose placement was the same as the time of application. Successful functionality was defined as a tag that was actively receiving and sending individual animal biometric data. Daily counts of pens entered by pen rider and caretakers, within each treatment group, were captured daily and reported herein. However, the time that a pen rider or caretaker spent observing and/or pulling cattle was not tracked in this study. Therefore, labor was not included in the subsequent economic analysis.

### Statistical Analyses

Pen was considered the experimental unit and the pen’s residing animals were the observational units for health and carcass traits (pen for feed performance) in this study. The day of enrollment was considered the block. An alpha of ≤ 0.05 was considered significant while an alpha of > 0.05 to ≤ 0.10 was considered a statistical trend. Analyses were performed using SAS software (SAS v9.4; Cary, NC). To evaluate the effect of treatment on health outcomes (pen-level proportion outcomes), Generalized Linear Mixed Models (GLMM) were fit using a binomial distribution and logit link. For evaluation of treatment effects on yield and quality grade outcomes (ordinal data), GLMMs were fit using a multinomial distribution and a cumulative logit link. Finally, to evaluate the effect of treatment on feed performance, other carcass metrics (e.g., hot carcass weight), and economic outcomes (continuous outcomes), Linear Mixed Models (LMM) were implemented. All models were fit with the Kenward-Roger degrees of freedom estimation procedure.

In all models, treatment main effects (i.e., BRD control strategy and BRD monitoring strategy) and the respective treatment interaction reflected the fixed effects of each model. Additionally, a random intercept for block was included to account for the design structure. If there was no significant interaction, but main effects were significant, pairwise comparisons were performed and adjusted by the Tukey method.

After enrollment of blocks 1 and 2, it was discovered the TBCP algorithm was performing incorrectly and under-identifying cattle at elevated risk for developing BRD based on internal validation procedures. Although the issue was resolved, retrospective analysis revealed many calves in those respective blocks were erroneously not administered BRD control. The authors concluded that only blocks 3 to 6 would initially be analyzed across the entire 3 × 2 factorial design for all health, performance, and carcass outcomes. If a statistical interaction was observed, the reported model-adjusted estimates would reflect only blocks 3 to 6. Conversely, if no interactions were present, model-adjusted estimates for the BRD control factor would reflect blocks 3 to 6 while the BRD detection factor would be reported utilizing all six blocks.

### Economic Analysis

A partial-budget for the BRD detection main effect (ADD vs PR) was completed by utilizing revenues and costs based on pen-level study outcomes and are reported in **Table 3**. Economic model revenue parameters included dressed cattle sales (adjusted for yield grade, quality grade, and light/heavy carcasses) and culled (railed) animals. Cost parameters within the model included cattle purchase price, animal health product cost at processing, feed/yardage costs, BRD antimicrobial treatment, and rendering fees for dead cattle. The difference between total revenue and costs (i.e., net return value) were calculated for each pen. The net return estimate reflects the outcome of the partial budget.

**Table 3.** Revenue and cost parameters among auction-market derived beef/beef-cross heifers in one Oklahoma feedlot monitored by either an automated disease detection (ADD) system or traditional pen-riding (PR) methods

Parameter	Value ($)	Source
**Revenue**		
Dressed harvest heifer price, $/45.4 kg	228.17	USDA AMS, 2023a
Quality grade adjustment, $/45.4 kg		USDA AMS, 2023b
Prime	20.18	
Choice	0.00	
Select	−24.63	
Standard	−36.02	
Yield grade adjustment, $/45.4 kg		
YG 1	5.00	
YG 2	2.04	
YG 3	0.00	
YG 4	−10.05	
YG 5	−13.83	
Carcass weight discount, $/45.5 kg		
181.4 – 226.8 kg	−35.20	
226.8 – 249.5 kg	−23.78	
249.5 – 272.2 kg	−3.48	
272.2 – 408.2 kg	0.00	
408.2 – 453.6 kg	−0.77	
453.6 – 476.3 kg	−2.73	
> 476.3 kg	−16.67	
Dressed cull price, $/45.5 kg		Horton et al, 2021 USDA AMS, 2023c
≥ 226.8 kg	Percentage of 128.22	
< 226.8 kg	Percentage of 124.45	
**Costs**		
Feeder purchase price, $/45.4 kg	149.17	Livestock Marketing Information Center, 2022
Ration, markup, and yardage, $/907.2 kg	375.91	Cattlefax, 2023
Initial processing, $/animal, or 45.4 kg	Modified-live viral vaccine (Bovilis Vista Once SQ^1^); $3.99/animal Multivalent clostridium toxoid (Bovilis Vision 7^1^); $0.84/animal Steroid implant (Revalor IH^1^); $3.34/animal Oral anthelmintic (Safeguard^1^)$0.25/45.4 kg Abortifacient (Estrumate^1^); $2.41/animal Injectable parasiticide (Dectomax^2^); $0.32/45.4 kg	
BRD control + BRD detection strategy; $/heifer	NC ± ADD; $0.00 + $9.28 PC ± ADD; Tildipirosin + $9.28 TBCP ± ADD; ± Tildipirosin + $2.25 + $9.28 NC ± PR; $0.00 + NA PC ± PR; Tildipirosin + NA TBCP ± PR; ± Tildipirosin + $2.25 + NA	
BRD treatment, $/heifer	First treatment: Florfenicol + Flunixin meglumine; $30.46 (ADD), $30.66 (PR) Second treatment: Enrofloxacin; $17.92 (ADD), $18.01 (PR) Third treatment: Oxytetracycline; $5.35 (ADD), $5.19 (PR)	
Mortality removal, $/heifer	38.27	Based on methods from Horton et al, 2023

^1^Merck & Co., Inc., Rahway, NJ, USA and its affiliates.

^2^Zoetis Animal health, Parsippany, NJ.

Dressed fed cattle sales were estimated based on data extracted from the United States Department of Agriculture Agricultural Marketing Service (USDA AMS) 5 Area Monthly Weighted Average Direct Slaughter Cattle—Negotiated report for the month of July 2022 (USDA AMS, 2023a). These data reflect the five areas (Texas/Oklahoma/New Mexico; Kansas; Nebraska; Colorado; Iowa/Minnesota feedlots) monthly weighted average *direct dressed delivered* heifer price for “all grades” ($228.17/ 45.4 kg). The base dressed revenue was adjusted by carcass-based premiums and discounts from the 5 Area Weekly Slaughter Cattle—Premiums and Discounts report (USDA AMS, 2023b). The price for each class was averaged over the reports for the weeks of: July 11^th^, 2022, July 18^th^, 2022, July 25^th^, 2022, and August 1^st^, 2022 (**Table 3**). To calculate the yield grade adjustments, the classes of “2.0 to 2.5” and “2.5 to 3.0” were averaged to create “Yield Grade 2” and classes “3.0 to 3.5” and “3.5 to 4.0” were averaged to created “Yield Grade 3”; all other classes were kept the same.

Revenue generated from cattle removed from the study (i.e., cull cattle) was calculated based on the estimated hot carcass weight (HCW) of railers, which was calculated based on their mean live weight using the formula from Tatum et al. (2012). The estimated HCW was categorized at greater than or equal to 226.8 kg or less than 226.8 kg, based on Horton et al. (2021), and used to determine a percentage of the price for dressed breaker cows nationally at the mid-point of the trial (December 2021 and January 2022). The average price of breaker cows in each category was calculated from the National Weekly Direct Cow and Bull Report—Negotiated Price (USDA AMS, 2023c) spanning the weeks that covered December 2021 and January 2022 (**Table 3**).

Total animal revenue sold on a dressed basis was calculated by multiplying the total pen HCW by the dressed harvest price and adjusting by the premium and discount prices. These prices were calculated by multiplying the premium/discount price with the total pen HCW that fell in each category (e.g., Prime price multiplied by Prime HCW). To calculate the revenue from cull cattle, the price per category (i.e., ≥ 226.8 kg or < 226.8 kg) was multiplied by the total pen estimated HCW in each category. To calculate the overall total revenue per pen, the revenue from animals sold on a dressed basis and from cull cattle was summed.

A feeder cattle purchase price of $149.17/49.5 kg was applied and based on the average monthly purchase price for 227 to 272 kg (500 to 600 lb) medium/large frame #1 feeder heifers from the Oklahoma City auction market from the Livestock Marketing Information Center (LMIC, 2022). The average monthly purchase price was calculated by averaging weekly prices for the months the animals were purchased and enrolled in the present study (August, September, and October 2021). The starting weight of the pen was multiplied by the feeder cattle purchase price to calculate the purchase price per pen.

The price for dry feed, feed markup, and yardage ($/907 kg [2,000 lb] dry feed) was calculated as the mean monthly cost using prices from August 2021 through July 2022 from the South Plains region (Cattle Fax, 2023). To calculate the overall ration, markup, and yardage price, the total feed consumed by each pen, deads and railers were included, was multiplied by the mean price of $375.91/ 907 kg dry feed.

Standardized initial processing costs, not inclusive of BRD control treatment, were used for all blocks, and product prices were sourced from PBS Animal Health on June 15, 2023 (www.pbsanimalhealth.com). The dosage to estimate price per animal was calculated based on manufacturer recommendations and the number of animals and starting weight of each respective pen. The product prices were: a modified-live viral vaccine at $3.99/animal (Bovilis Vista Once SQ, Merck & Co., Inc., Rahway, NJ, USA and its affiliates), a multivalent clostridium toxoid at $0.84/animal (Bovilis Vision 7, Merck & Co., Inc., Rahway, NJ, USA and its affiliates), a steroid implant at $3.34/animal (Revalor IH, Merck & Co., Inc., Rahway, NJ, USA and its affiliates), an oral anthelmintic at $0.25/45.4 kg (Safeguard, Merck & Co., Inc., Rahway, NJ, USA and its affiliates), an injectable parasiticide at $0.32/45.4 kg (Dectomax, Zoetis Animal health, Parsippany, NJ), and an abortifacient at $2.41/animal (Estrumate, Merck & Co., Inc., Rahway, NJ, USA and its affiliates).

To account for the cost of BRD control, the price of $4.70/45.4 kg (Zuprevo, Merck & Co., Inc., Rahway, NJ, USA and its affiliates) was differentially applied according to the BRD control treatment group. Pens assigned as the NC received no BRD control antimicrobial and thus no corresponding cost. For pens assigned to PC, where all animals received the BRD control antimicrobial, the starting weight of the pen was divided by 45.4 kg and multiplied by the price per 45.4 kg. For pens assigned to TBCP, the average animal starting weight was multiplied by the number of animals identified to receive the BRD control antimicrobial; this pen-level weight was divided by 45.4 kg and then multiplied by the price per 45.4 kg. Additionally, each calf within the TBCP incurred an additional use cost of $2.25/hd. The tags used within the ADD system are designed for reuse to optimize cost/head. In practice, the most practical timepoint to collect that hardware is at the time of reimplant. For the purposes of this study, all tags were left in for the study duration. However, the economic model was designed with reimplant removal in mind. Therefore, lots in which the ADD technology was used to monitor health, a cost of $9.28/animal was added which reflected a $29 tag, three uses per year across a two-year battery life (i.e., 6 total uses), a $4.29 reactivation fee for each use after the first use, and fixed costs of additional hardware (e.g., antenna). As stated above, labor (cost outlay or cost savings) was not accounted for within the economic analysis. Additionally, no financial risk mitigation strategies were assumed within the economic model. Therefore, the total processing cost per animal included the fixed costs dose-dependent products plus the costs of the weight-based products for initial processing and BRD control and detection (**Table 3**).

Morbidity costs per animal differed for the disease detection treatments, as the average moribund animal weight was different per group. The price per treatment per animal for the different disease detection treatments are reported in **Table 3**. The price includes a $2 chute charge. Treatment costs were multiplied by the number of animal treatments in each pen. An estimated cost for rendering a dead animal was used for all dead animals, at $38.27 based on the methods of Horton et al., (2023).

## RESULTS

### Descriptive Statistics

A total of 2,542 heifer calves averaging 257.3 kg (range, 169.6 kg to 381 kg) at arrival were enrolled over a six-week period during the latter third quarter and early fourth quarter of 2021. Start dates for each block were as follows; Block 1 = August 30, 2021; Block 2 = September 6, 2021; Block 3 = September 13, 2021; Block 4 = September 27, 2021; Block 5 = September 20, 2021; and Block 6 = October 4, 2021. Across these enrollment timeframes, the study population was composed of calves procured from 5 to 9 different salebarns within Texas and Oklahoma. At the time of enrollment, one calf was not included in the study population due to lack of sufficient ear tissue. Additionally, four calves were identified post-enrollment as being persistently infected with bovine viral diarrhea virus. These calves were removed and quarantined from the study population within 48 hours of study enrollment. Across the entire study population (independent of treatment group) from Day 0 to 60, 41.5% of the study population (N = 1055) were diagnosed and treated for BRD at least one time. Additionally, 4.1% (N = 104) and 4.4% (N = 113) died or were removed, respectively. From Day 0 to closeout (224 d), 44.5% of the study population (N = 1,131) were diagnosed and treated for BRD at least one time. Additionally, 6.5% (N = 165) and 7.1% (N = 180) died or were removed, respectively. Among the lots allocated to the TBCP (Treatments 5 and 6, respectively; **Table 1**), the average reduction in BRD control antimicrobial use (compared to the PC) was 25% (range: 18% - 33%). Upon displaying clinical signs of depression, three calves in the ADD group were detected by caretakers that had not been alerted by the ADD system on those respective days. Upon further inspection, it was determined by feedlot personnel that these calves had previously been alerted by the ADD system, treated accordingly, were currently in a post-treatment interval, and therefore not eligible for therapy. Those calves were subsequently returned to their pens of origin without further therapy at that time.

### Health Results at 60 Days on Feed

The 60-day model-adjusted means and standard errors of the means for health outcomes are displayed in **Table 4**. No significant (*P *> 0.05) interactions between the BRD control and disease detection treatments were observed at this timepoint. The BRD morbidity estimate among cattle allocated to the PC (36.6% [± 2.5%]) was reduced (*P *≤ 0.05) compared to the NC (42.5% [± 2.5%]) and the TBCP (51.0% [± 2.6%]). Overall mortality (*P *= 0.09) and overall fallouts (*P *= 0.08) tended to increase in the NC and TBCP compared to the PC.

**Table 4.** Model-adjusted^1^ means (standard error of the means) for the 60-day health outcomes within a 3 × 2 factorial study composed of auction-market derived beef/beef-cross heifers in one Oklahoma feedlot. Cattle received one of three different BRD control programs and were monitored by one of two BRD detection strategies. Due to technical issues with the TBCP technology, only blocks 3 to 6 were used to analyze the BRD control strategy outcomes. If no interaction was observed, all blocks were used to estimate disease detection strategy outcomes. Otherwise, blocks 3 to 6 were used for both factors

Parameter	BRD control strategy ^2^				Disease detection strategy ^3^			BRD control strategy x Disease detection strategy
	NC	PC	TBCP	*P*-value	ADD	PR	*P*-value	*P*-value
Pen count/Animal count	12/847	12/847	12/848	NA^4^	18/1,270	18/1,274	NA	NA
Arrival weight, kg ^5^	259.1 (2.6)	255.6 (2.6)	259.5 (2.6)	0.08	258.4 (1.7)	256.3 (1.7)	0.09	0.21
BRD morbidity, %	42.5 (2.5) ^a^	36.6 (2.5) ^b^	51.0 (2.6) ^a^	<0.01	42.7 (2.3)	41.8 (2.3)	0.63	0.08
Day at 1st BRD treatment	16.0 (1.0)	15.4 (1.0)	13.0 (1.0)	0.29	14.0 (0.8)	16.1 (0.8)	0.03	0.69
Rectal temperature at 1st treatment, °C	40.2 (0.1)	40.3 (0.1)	40.4 (0.1)	0.14	40.1 (0.1)	40.5 (0.1)	<0.01	0.36
BRD 2nd treatment, %	55.1 (3.8)	57.7 (4.0)	53.4 (3.6)	0.62	53.3 (3.3)	52.3 (3.3)	0.74	0.26
Days between 1^st^ and 2^nd^ BRD treatment	11.7 (1.2)	14.0 (1.2)	13.6 (1.2)	0.32	11.5 (0.9)	15.2 (0.9)	<0.01	0.97
BRD 3rd treatment, %	53.5 (4.5)	54.5 (4.7)	54.4 (4.2)	0.98	57.5 (3.6)	45.7 (3.7)	0.01	0.69
Time between 1^st^ and 3^rd^ BRD treatment, days	16.2 (1.1) ^a^	19.6 (1.1) ^b^	18.8 (1.1)^a,b^	0.05	18.4 (1.4)	20.2 (1.4)	0.16	0.18
BRD treatment success, % ^6^	36.1 (3.9)	38.1 (4.1)	42.1 (3.8)	0.34	43.6 (3.4)	38.0 (3.3)	0.07	0.21
Overall mortality, %	4.9 (1.4)	2.4 (0.9)	4.0 (1.2)	0.09	3.1 (0.7)	4.6 (1.0)	0.05	0.65
BRD mortality, %	4.5 (1.4)	2.5 (0.9)	3.8 (1.2)	0.15	2.9 (0.7)	4.4 (0.9)	0.06	0.69
BRD case fatality, %	4.5 (2.1)	4.2 (2.0)	6.7 (2.7)	0.34	6.0 (1.9)	6.0 (1.9)	0.98	0.61
Day at 1^st^ BRD treatment among dead calves	8.2 (3.3)	13.6 (3.5)	16.0 (3.3)	0.26	15.5 (2.1)	13.7 (2.1)	0.53	0.29
BRD pen-deads, % ^7^	Model did not converge ^8^							
Overall removals, % ^9^	6.7 (1.1)	5.3 (1.0)	5.6 (1.0)	0.57	4.4 (0.7)	6.0 (0.9)	0.08	0.85
BRD removals, %	6.5 (1.1)	4.8 (0.9)	5.6 (1.0)	0.65	4.2 (0.7)	5.7 (0.8)	0.10	0.88
Overall fallouts, % ^10^	11.8 (2.1)	7.8 (1.6)	9.6 (1.8)	0.08	7.4 (1.2)	10.6 (1.6)	<0.01	0.96
BRD fallouts, %	9.0 (1.7)	6.5 (1.4)	9.3 (1.8)	0.16	6.8 (1.1)	8.6 (1.3)	0.11	0.92

^1^Mixed models with a random effect to account for the lack of independence among blocks.

^2^Three BRD control strategies were evaluated: Negative Control (NC), Positive Control (PC; 100% of treatment group received the BRD control antimicrobial which reflects traditional management), or a Targeted BRD Control Program (TBCP; individual animal prediction of BRD risk generated by a novel hardware/software system capturing cardiopulmonary data subsequently analyzed by machine learning techniques).

^3^Two disease detection methods were evaluated: an automated disease detection system (ADD; a novel hardware/software system designed to continuously capture animal temperature and activity and whose data is analyzed by machine learning techniques) and traditional pen riding (PR) practices which reflect conventional management.

^4^NA: Not Applicable.

^5^If a statistical difference was observed in incoming body weight treatment groups, this variable was utilized as a covariate for the remainder of the respective models.

^6^Calves treated for BRD that did not require additional BRD therapy, were not removed, and did not die.

^7^This estimate reflects calves that were found dead without prior history of BRD treatment.

^8^Model did not converge: Lack of sufficient observations from Day 0 to 60 to generate a model-adjusted estimate.

^9^This estimate reflects non-mortality removals (e.g., chronic BRD, non-BRD syndromes, etc.).

^10^Mortalities + removals.

^a, b^Different superscripts within a row denote significant differences when alpha ≤ 0.05 and adjusted for multiple comparisons.

By 60 DOF, cattle monitored by the ADD technology were identified and treated sooner (*P *= 0.03;14.0 d [± 0.8 d] vs 16.1 d [± 0.8 d]) for cattle in the PR group, and displayed lower (*P *< 0.01) rectal temperatures at first BRD treatment (40.1°C [± 0.1°C] vs 40.5°C [± 0.1°C]) compared to the PR group, respectively (**Table 4**). No differences (*P > *0.05) in the cumulative incidence of BRD morbidity or BRD retreatment were observed between the two groups. However, the ADD group displayed a reduction (*P *< 0.01) in days between the first and second BRD treatment compared to the PR group (11.5 d [± 0.9 d] vs 15.2 d [± 0.9 d], respectively) and an increase (*P *= 0.01) in BRD third treatments compared to the PR group (57.5% [± 3.6%] vs 45.7% [± 3.7%], respectively). However, the ADD group displayed a reduction (*P *= 0.05) in overall death loss (3.1% [± 0.7%]) compared to the PR group (4.6% [± 1.0%]) and a reduction (*P *< 0.01) in overall fallouts (mortalities + removals; 7.4% [± 1.2%]) compared to the PR group (10.6% [± 1.6%]) at the 60-day timeframe (**Table 4**).

### Closeout Health Outcomes

The model-adjusted means and standard errors of the means for health outcomes at closeout (224 d on feed) are displayed in **Table 5**. No significant (*P *> 0.05) interactions between the BRD control and disease detection treatments were observed at this timepoint. However, statistical tendencies (*P* = 0.09) were observed in the BRD morbidity and BRD pen-dead interactions. At closeout, the PC displayed a reduction (*P *< 0.01) in BRD morbidity (38.2% [± 2.2%]) compared to the NC (44.9% [2.3%]) and the TBCP (52.2% [± 2.3%]). No differences in BRD morbidity were observed between the NC and TBCP. Interestingly, the TBCP observed a reduction (*P *≤ 0.01) in the time to first BRD treatment (14.2 d [± 1.4 d]) compared to the NC (19.8 d [± 1.8 d]) and PC (18.4 d [± 1.4 d]). The PC displayed a reduction (*P *= 0.02) in overall and BRD mortality (4.2% [± 1.0%] and 3.3% [± 1.0%], respectively) compared to the NC (8.7% [1.5%] and 7.1% [± 1.6%], respectively). Likewise, the PC displayed a reduction (*P *= 0.02) in overall and BRD fallouts (11.1% [± 1.7%] and 9.6% [± 1.6%], respectively) compared to the NC (17.0% [± 2.2%] and 15.4% [2.2%], respectively). No remaining health outcomes were observed to be different (*P *> 0.05) at the timepoint of closeout among the BRD control strategies (**Table 5**). However, the reader should note that although not statistically significant, large numerical differences were observed (in favor of PC) between the PC and TBCP among the overall mortality, BRD mortality, overall fallouts, and BRD fallout parameters.

**Table 5.** Model-adjusted^1^ means (standard error of the means) for the closeout (224 d on feed) health outcomes within a 3 × 2 factorial clinical trial composed of auction-market derived beef/beef-cross heifers in one Oklahoma feedlot. Cattle received one of three different bovine respiratory disease (BRD) control programs and were monitored by one of two disease detection strategies. Due to technical issues with the TBCP technology, only blocks 3 to 6 were used to analyze the BRD control strategy outcomes. If no interaction was observed, all blocks were used to estimate disease detection strategy outcomes. Otherwise, blocks 3 to 6 were used for both factors

Parameter	BRD control strategy ^2^				Disease detection strategy ^3^			BRD control strategy × Disease detection strategy
	NC	PC	TBCP	*P*-value	ADD	PR	*P*-value	*P*-value
Pen count/Animal count	12/847	12/847	12/848	NA^4^	18/1,270	18/1,274	NA	NA
Arrival weight, kg^5^	259.1 (2.5)	255.6 (2.5)	259.5 (2.5)	0.08	258.4 (1.7)	256.3 (1.7)	0.09	0.21
BRD morbidity, %	44.9 (2.3) ^a^	38.2 (2.2) ^b^	52.2 (2.3) ^a^	<0.01	44.0 (2.2)	44.1 (2.2)	0.94	0.09
Day on feed at 1st BRD treatment	19.8 (1.5) ^a^	18.4 (1.4) ^a^	14.2 (1.4) ^b^	< 0.01	16.3 (1.1)	20.0 (1.1)	<0.01	0.76
Rectal temperature at 1st treatment, °C	40.3 (0.1)	40.3 (0.1)	40.4 (0.1)	0.52	40.1 (0.1)	40.6 (0.1)	<0.01	0.62
BRD 2nd treatment, %	55.1 (3.6)	58.5 (3.8)	55.2 (3.4)	0.68	54.5 (2.7)	54.2 (2.7)	0.91	0.25
Time between 1^st^ and 2^nd^ BRD treatment, days	12.3 (1.6) ^b^	16.7 (1.6) ^a^	15.2 (1.6) ^a,b^	0.01	13.3 (1.9)	19.2 (1.9)	<0.01	0.85
BRD 3rd treatment, %	60.2 (4.1)	57.2 (4.4)	60.0 (3.8)	0.87	58.8 (3.5)	54.0 (3.6)	0.25	0.30
Time between 1^st^ and 3^rd^ BRD treatment, days	20.0 (3.1)	23.2 (3.1)	26.8 (3.1)	0.24	22.4 (2.5)	26.2 (2.5)	0.19	0.77
BRD treatment success, %^6^	37.9 (3.8)	36.6 (3.9)	40.1 (3.6)	0.68	42.7 (2.9)	37.7 (2.8)	0.10	0.45
Overall mortality, %	8.7 (1.5) ^a^	4.2 (1.0) ^b^	6.7 (1.2) ^a,b^	0.02	5.7 (0.8)	6.7 (0.9)	0.27	0.22
BRD mortality, %	7.1 (1.6) ^a^	3.3 (1.0) ^b^	6.3 (1.4) ^a,b^	0.02	4.7 (0.8)	5.7 (1.0)	0.27	0.27
BRD case fatality, %	10.0 (2.7)	6.2 (2.1)	10.4 (2.7)	0.23	9.7 (2.0)	8.7 (1.9)	0.58	0.30
Day on feed at 1^st^ BRD treatment. among BRD mortalities	13.9 (4.6)	10.9 (4.9)	11.0 (4.6)	0.80	10.4 (3.3)	17.7 (3.3)	0.06	0.36
Day on feed at death	55.5 (13.9)	54.0 (13.9)	71.5 (13.)	0.42	61.3 (9.8)	60.1 (9.6)	0.91	0.65
BRD pen-deads, %^7^	1.3 (0.7)	0.9 (0.4)	0.7 (0.4)	0.72	0.4 (0.2)	1.7 (0.4)	0.01	0.09
Overall removals, %^8^	8.2 (1.3)	6.8 (1.1)	8.2 (1.2)	0.56	5.5 (0.8)	8.3 (1.0)	0.01	0.85
BRD removals, %	6.7 (1.1)	5.2 (0.9)	6.4 (1.0)	0.66	4.6 (0.7)	6.6 (0.8)	0.04	0.91
Overall fallouts, %^9^	17.0 (2.2) ^a^	11.1 (1.7) ^b^	14.7 (2.0) ^a,b^	0.02	11.1 (1.4)	15.2 (1.7)	<0.01	0.55
BRD fallouts, %	15.4 (2.2) ^a^	9.6 (1.6) ^b^	13.2 (2.0) ^a,b^	0.02	9.6 (1.2)	13.9 (1.6)	<0.01	0.68

^1^Mixed models with a random effect to account for the lack of independence among blocks.

^2^Three BRD control strategies were evaluated: Negative Control (NC), Positive Control (PC; 100% of treatment group received the BRD control antimicrobial which reflects traditional management), or a Targeted BRD Control Program (TBCP; individual animal prediction of BRD risk generated by a novel hardware/software system capturing cardiopulmonary data subsequently analyzed by machine learning techniques).

^3^Two disease detection methods were evaluated: an automated disease detection system (ADD; a novel hardware/software system designed to continuously capture animal temperature and activity and whose data is analyzed by machine learning techniques) and traditional pen riding (PR) practices which reflect conventional management.

^4^NA: Not Applicable.

^5^If a statistical difference was observed in incoming body weight treatment groups, this variable was utilized as a covariate for the remainder of the respective models.

^6^Calves treated for BRD that did not require additional BRD therapy, were not removed, and did not die.

^7^This estimate reflects calves that were found dead without prior history of BRD treatment.

^8^This estimate reflects non-mortality removals (e.g., chronic BRD, non-BRD syndromes, etc.).

^9^Mortalities + removals.

^a, b^Different superscripts within a row denote significant differences when alpha ≤ 0.05 and adjusted for multiple comparisons.

At the time of closeout, the ADD group displayed a reduction (*P *< 0.01) in both days on feed to first BRD treatment (16.3 d [± 1.1 d] vs 20.0 d [± 1.1 d]) and rectal temperature at first BRD treatment (40.1°C [± 0.1°F] vs 40.6°F [± 0.1°C]) compared to the PR group, respectively. No differences (*P *> 0.05) were observed between the two groups for cumulative BRD morbidity or BRD retreatment incidence. However, calves monitored by the ADD technology were detected sooner (*P* < 0.01) between the first and second BRD treatments (13.3 d [± 1.9 d]) compared to calves monitored by the PR (19.2 d [± 1.9 d]). No differences (*P* > 0.05) were observed in BRD third treatments or BRD treatment success between the two monitoring modalities. No differences (*P* > 0.05) were observed in overall mortality or BRD mortality risk between the two groups. However, calves monitored by the ADD system displayed a reduction (*P *= 0.01) in BRD pen-deads (i.e., cattle dying without diagnosis and therapy) compared to the PR (0.4% [± 0.2%] vs 1.7% [± 0.4%], respectively). Calves monitored by the ADD system displayed a reduction in overall (*P *= 0.01) and BRD (*P = *0.04) removals (5.5% [± 0.8%] and 4.6% [± 0.7%], respectively) compared to PR calves (8.3% [± 1.0%] and 6.6% [± 0.8%], respectively). Likewise, calves monitored by the ADD system experienced a reduction (*P < *0.01) in overall and BRD fallouts (11.1% [± 1.4%] and 9.6% [± 1.2%], respectively) compared to the PR group (15.2% [± 1.7%] and 13.9% [1.6%], respectively).

### Performance Outcomes

Closeout performance outcomes are displayed in **Table 6**. Interactions were observed between the BRD control and disease detection treatments for daily dry-matter intake (DMI) and gain:feed (G:F; deads and removals not included in the estimate). However, after adjusting for multiple comparisons, no significant (*P > *0.05) differences were observed between the interactive treatments. Among the BRD control treatments the PC displayed an increase (*P *= 0.03) in average daily gain ([ADG], dead and removed cattle included in the estimate; 1.09 kg/d [± 0.05 kg/d]) compared to the TBCP (0.98 kg/d [± 0.05 kg/d]). However, the NC estimate (1.00 kg/d [± 0.05 kg/d]) was not observed to be different (*P > *0.05) compared to either the PC or TBCP. Likewise, the PC displayed an increase (*P* = 0.05) in G:F (dead and removed cattle included in the estimate; 0.178 [± 0.004]) compared to the TBCP (0.139 [± 0.004]). The analogous NC estimate (0.144 [± 0.004]) was not different (*P > *0.05) compared to either the PC or TBCP. No other performance outcomes displayed significance (*P *> 0.05) among the BRD control treatments.

**Table 6.** Model-adjusted^1^ means (standard error of the means) for the closeout (224 d on feed) feed performance outcomes within a 3 × 2 factorial clinical trial composed of auction-market derived beef/beef-cross heifers in one Oklahoma feedlot. Cattle received one of three different bovine respiratory disease (BRD) control programs and were monitored by one of two disease detection strategies. Due to technical issues with the TBCP technology, only blocks 3 to 6 were used to analyze the BRD control strategy outcomes. If no interaction was observed, all blocks were used to estimate disease detection strategy outcomes. Otherwise, blocks 3 to 6 were used for both factors

Parameter	BRD control strategy ^2^				Disease detection strategy ^3^			BRD control strategy × Disease detection strategy
	NC	PC	TBCP	*P*-value	ADD	PR	*P*-value	*P*-value
Pen count/Animal count	12/847	12/847	12/848	NA^4^	18/1,270	18/1,274	NA	NA
Arrival weight, kg	259.1 (2.6)	255.6 (2.6)	259.5 (2.6)	0.08	258.4 (1.7)	256.3 (1.7)	0.09	0.21
Final body weight (D/R^5^-out), kg	548.8 (5.1)	548.3 (5.1)	548.7 (5.1)	0.99	546.1 (4.9)	551.0 (4.9)	0.09	0.28
Weight gain (D/R-out), kg	286.6 (5.4)	288.9 (5.4)	291.0 (5.4)	0.31	283.1 (4.2)	287.0 (4.2)	0.07	0.28
ADG^6^ (D/R-out), kg/day	1.27 (0.02)	1.28 (0.02)	1.29 (0.02)	0.31	1.27 (0.01)	1.29 (0.01)	0.07	0.48
ADG (D/R-in), kg/day	1.00 (0.05) ^a,b^	1.09 (0.05) ^a^	0.98 (0.05) ^b^	0.03	1.06 (0.02)	1.00 (0.02)	0.06	0.28
Daily DMI^7^, kg/heifer	7.2 (0.1)	7.2 (0.1)	7.2 (0.1)	0.96	7.1 (0.1)	7.3 (0.1)	0.04	0.02 ^8^
G:F^9^ (D/R-out), kg of gain/kg of feed	0.178 (0.002)	0.178 (0.002)	0.180 (0.002)	0.80	0.179 (0.002)	0.179 (0.002)	1.00	0.02 ^8^
G:F (D/R-in), kg of gain/kg of feed	0.144 (0.004) ^a,b^	0.155 (0.004) ^a^	0.139 (0.004) ^b^	0.05	0.149 (0.004)	0.139 (0.004)	< 0.01	0.21
Final body weight per heifer placed, kg	463.5 (9.3)	489.0 (9.3)	456.9 (9.3)	0.06	479.3 (6.1)	461.1 (6.1)	0.03	0.26

^1^Mixed models with a random effect to account for the lack of independence among blocks.

^2^Three BRD control strategies were evaluated: Negative Control (NC), Positive Control (PC; 100% of treatment group received the BRD control antimicrobial which reflects traditional management), or a Targeted BRD Control Program (TBCP; individual animal prediction of BRD risk generated by a novel hardware/software system capturing cardiopulmonary data subsequently analyzed by machine learning techniques).

^3^Two disease detection methods were evaluated: an automated disease detection system (ADD; a novel hardware/software system designed to continuously capture animal temperature and activity and whose data is analyzed by machine learning techniques) and traditional pen riding (PR) practices which reflect conventional management.

^4^NA: Not Applicable.

^5^D/R: Deads and removals.

^6^ADG: Average daily gain.

^7^DMI: Dry-matter intake.

^8^Despite a *P*-value ≤ 0.05, no statistical differences were observed among interactive treatments when adjusting for multiple comparisons (Tukey method).

^9^G:F: Gain:Feed.

^a, b^Different superscripts within a row denote significant differences when alpha ≤ 0.05 and adjusted for multiple comparisons.

Between disease detection treatments, an increase (*P *= 0.04) in dry-matter intake was observed between the PR and the ADD system (7.3 kg/d [± 0.1 kg/d] vs 7.1 kg/d [± 0.1 kg/d], respectively). However, an increase (*P *< 0.01) in G:F (dead and removed cattle included in the estimate) was observed among the ADD group compared to the PR group (0.149 kg gain/kg feed [± 0.004 kg gain/kg feed] vs. 0.139 kg gain/kg feed [± 0.004 kg gain/kg feed], respectively). Additionally, final body weight per head originally placed within the ADD group was greater (*P *= 0.03) compared to the PR (479.3 kg [± 6.1 kg] vs 461.1 kg [± 6.1 kg]). No further outcomes displayed statistically significant (*P *> 0.05) findings.

### Carcass Outcomes

Carcass outcomes are displayed in **Table 7**. Backfat and calculated yield grade were higher (*P* < 0.05) for NC (0.61 inches [± 0.03 inches] and 3.3 [± 0.1], respectively) and PC (0.61 inches [± 0.03 inches] and 3.3 [± 0.01], respectively) compared to TBCP (0.57 inches [± 0.03 inches] and 3.2 [± 0.1], respectively). Total hot carcass weight (HCW) per pen of PC (22,073 kg/pen [± 539 kg/pen) was greater (*P *= 0.05) than the NC (20,623 kg/pen [± 539 kg/pen]) and TBCP (20755 kg/pen [± 539 kg/pen]). Additionally, the PC displayed an increase (*P *= 0.04) in the average total HCW per head originally placed (308.2 kg/heifer placed [± 6.1 kg/heifer placed]) compared to the NC (288.4 kg/heifer placed [± 6.1 kg/heifer placed]). No differences (*P *> 0.05) were observed in the remaining carcass outcomes among the BRD control treatment groups.

**Table 7.** Model-adjusted^1^ means (standard error of the means) for the closeout (224 d on feed) carcass outcomes within a 3 × 2 factorial clinical trial composed of auction-market derived beef/beef-cross heifers in one Oklahoma feedlot. Cattle received one of three different BRD control programs and were monitored by one of two BRD detection strategies. Due to technical issues with the TBCP technology, only blocks 3 to 6 were used to analyze the BRD control strategy outcomes. If no interaction was observed, all blocks were used to estimate disease detection strategy outcomes. Otherwise, blocks 3 to 6 were used for both factors

Parameter	BRD control strategy ^2^				Disease detection strategy^3^			BRD control strategy × Disease detection strategy
	NC	PC	TBCP	*P*-value	ADD	PR	*P*-value	*P*-value
Pen count/carcass count	12/710	12/752	12/722	NA^4^	18/1,118	18/1,070	NA	NA
Hot Carcass weight^5^ (D/R^6^ out), kg	351.7 (4.9)	349.8 (4.9)	348.4 (4.9)	0.46	344.0 (4.1)	347.3 (4.1)	0.07	0.20
Dressing %	63.3 (0.3)	63.4 (0.3)	63.3 (0.3)	0.70	63.1 (0.2)	63.2 (0.2)	0.38	0.62
Ribeye area, square inches	13.5 (0.2)	013.5 (0.2)	13.5 (0.2)	0.99	13.2 (0.2)	13.3 (0.2)	0.25	0.56
Marbling	471.9 (10.1)	470.1 (10.1)	456.9 (10.1)	0.23	458.2 (8.0)	459.6 (8.0)	0.84	0.21
Backfat, inches	0.61 (0.03) ^a^	0.61 (0.03) ^a^	0.57 (0.03) ^b^	0.03	0.58 (0.02)	0.58 (0.02	0.79	0.49
Calculated Yield Grade	3.3 (0.1) ^a^	3.3 (0.1) ^a^	3.2 (0.1) ^b^	0.05	3.2 (0.1)	3.2 (0.1)	0.85	0.84
Liver, % normal	64.0 (5.7)	64.0 (5.7)	59.7 (5.7)	0.77	67.0 (3.7)	65.9 (3.7)	0.76	0.84
Yield Grade, %	% of treatment group (count)			0.06	% of treatment group (count)		0.76	0.96
1	4.5% (32)	3.5% (26)	4.5% (32)	N = 2,167	3.5% (39)	4.8% (51)	N = 2,167	N = 2,167
2	34.5% (243)	32.9% (245)	38.3% (275)		35.5% (393)	34.9% (370)		
3	44.7% (315)	45.7% (340)	43.3% (311)		45.9% (508)	43.2% (458)		
4	14.0% (99)	16.5% (123)	13.4% (96)		13.9% (154)	15.5% (164)		
5	14.0% (16)	16.5% (10)	13.4% (4)		1.1% (12)	1.7% (18)		
Quality Grade, %	% of treatment group (count)			0.16	% of treatment group (count)		0.30	0.10
Prime	3.0% (21)	2.8% (21)	2.1% (15)	N = 2,167	2.3% (25)	3.0% (32)	N = 2,167	N = 2,167
Choice	65.4% (461)	71.4% (531)	64.8% (465)		69.3% (766)	65.1% (691)		
Select	31.2% (220)	25.4% (189)	33.0% (237)		28.2% (312)	31.5% (334)		
Other	0.4% (3)	0.4% (3)	0.1% (1)		0.3% (3)	0.4% (4)		
Avg. total HCW per pen, kg	20,623 (539) ^a^	22,073 (539) ^b^	20,755 (539) ^a^	0.05	21,201 (380)	20,498 (380)	0.07	0.38
Avg. total HCW per heifer originally placed, kg	288.4 (6.1) ^a^	308.2 (6.1) ^b^	289.8 (6.1) ^a,b^	0.04	300.9 (4.2)	289.7 (4.2)	0.03	0.31

^1^Mixed models with a random effect to account for the lack of independence among blocks.

^2^Three BRD control strategies were evaluated: Negative Control (NC), Positive Control (PC; 100% of treatment group received the BRD control antimicrobial which reflects traditional management), or a Targeted BRD Control Program (TBCP; individual animal prediction of BRD risk generated by a novel hardware/software system capturing cardiopulmonary data subsequently analyzed by machine learning techniques).

^3^Two disease detection methods were evaluated: an automated disease detection system (ADD; a novel hardware/software system designed to continuously capture animal temperature and activity and whose data is analyzed by machine learning techniques) and traditional pen riding (PR) practices which reflect conventional management.

^4^NA: Not Applicable.

^5^HCW: Hot carcass weight.

^6^D/R: Deads and removals.

^a, b^Different superscripts within a row denote significant differences when alpha ≤ 0.05 and adjusted for multiple comparisons.

Between the disease detection treatments, the average HCW per head originally placed was greater (*P *= 0.03) for the ADD group compared to the PR group (300.9 kg/heifer placed [± 4.2 kg/heifer placed] vs. 289.7 kg/heifer placed [± 4.2 kg/heifer placed], respectively). No differences (*P *> 0.05) were observed between the disease detection treatment groups for the remaining carcass metrics.

### Tag Retention and Functionality

Tag retention and tag functionality was monitored throughout the study and were consolidated into one estimate. By Day 60, tag retention/functionality was observed to be 98.3%. At the time of closeout, the tag retention/functionality was observed to be 97%.

### Labor Observations

**Figure 1.**
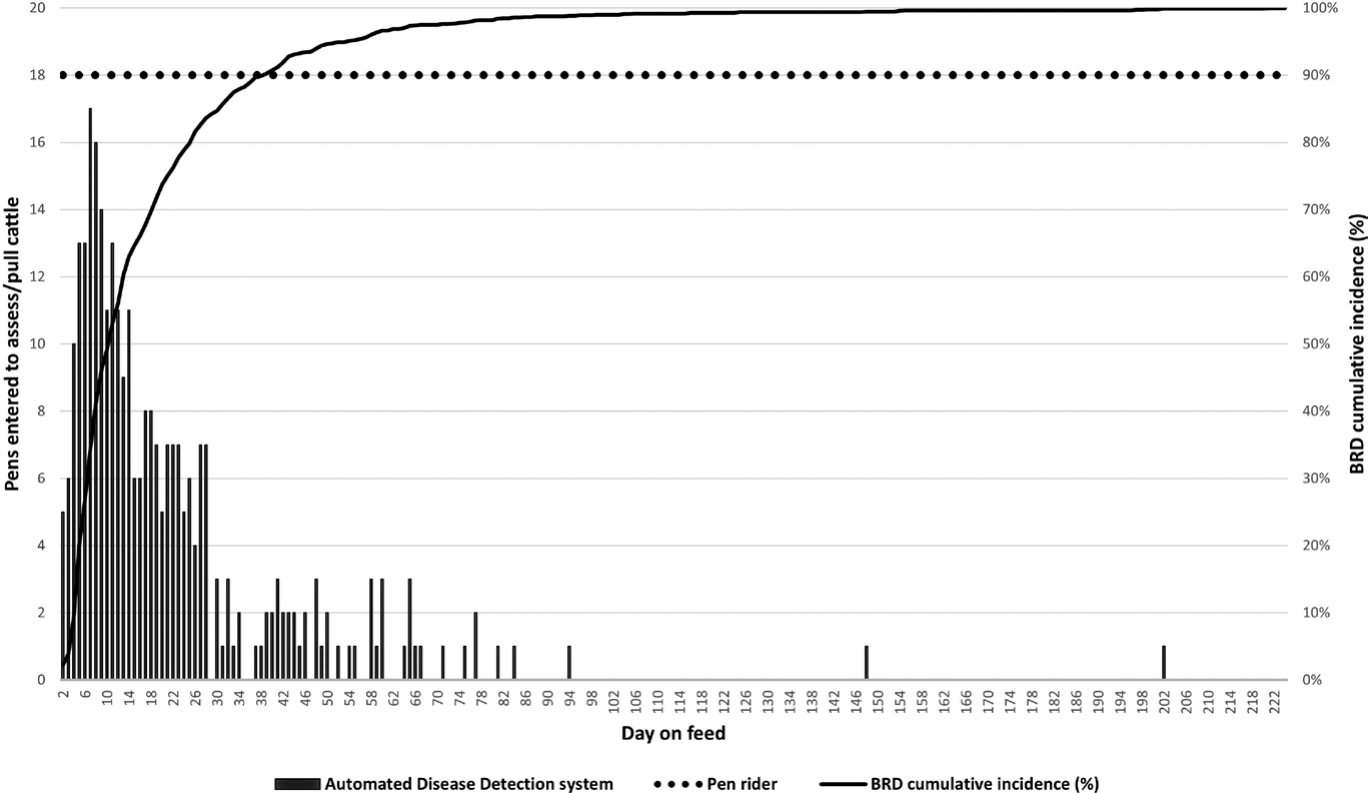
Descriptive counts of feedlot pens entered each day by caretakers assigned to retrieve calves alerted by an automated disease detection system (black vertical bars) or by individuals monitoring pens by traditional means (pen riders; dotted line). The cumulative BRD incidence (black line; right y-axis) is displayed for context.

As stated above, labor metrics were not incorporated into the economic analysis, nor were they measured in terms of time spent either observing and pulling cattle (PR treatment group) or retrieving cattle alerted by the ADD technology (ADD treatment group). The descriptive results of pen counts entered per day are displayed in **Figure 1**. Personnel assigned to the PR group entered each pen (N = 18) every day to visually assess animals in a traditional manner. Conversely, caretakers assigned to the ADD group entered several pens daily during the early weeks of the study which reflected the timeframe of the rapidly increasing incidence of BRD (**Figure 1**). However, the daily number of ADD pens that were entered by caretakers reduced over time (**Figure 1**).

### Economic Outcomes for the ADD System

Cattle monitored by the PR returned an average of $29.50 less per heifer compared to cattle monitored by the ADD technology (95% CI, $-60.28 to $1.27/heifer; *P* = 0.06). As stated above, these estimates do not incorporate market risk mitigation strategies and are independent of labor input.

## DISCUSSION

Bovine respiratory disease is a highly prevalent and economically devastating syndrome in post-weaned beef cattle. Although it can be speculated that incoming groups have been exposed to risk factors previously associated with developing BRD, specific health histories are often unknown. These obstacles are further compounded as current BRD detection modalities (i.e., human observation) are variable and subsequently inaccurate. Finally, these preexisting challenges are further intensified by difficulty in locating and retaining feedlot labor resources.

Traditionally, BRD control programs (i.e., metaphylaxis) have been designed and implemented to address the challenges of 1) unknown disease status at the time of arrival, 2) accurate BRD detection, and 3) labor optimization. Numerous studies have not only reported clinical and performance benefits of multiple BRD control programs but also the economic value of this management practice (Booker et al., 2007; Van Donkersgoed et al., 2008; Dennis et al., 2018).

Despite the value BRD control programs provide, there are concerns by the consumer regarding antimicrobial use in food animals (Baptiste and Kyvsgaard, 2017; FDA, 2018; CDC, 2019). Therefore, alternatives to traditional BRD control programs may be of value. In a prior study, the TBCP technology displayed no differences in health, performance, and carcass metrics when compared directly to a traditional metaphylaxis program, but reduced metaphylactic antimicrobial use up to 43% (Nickell et al., 2021). However, based on low mortality outcomes among negative controls, the disease pressure in that study was considered minimal (Nickell et al., 2021). In the current study, removing blocks 1 and 2 reduced statistical power by removing experimental units from analysis. Nonetheless, disease pressure in the current study was substantially greater thereby offering a more robust test of the TBCP system. The elevated disease pressure did appear to translate into less animals predicted to be at lower risk of disease as only 25% of animals (on average) in the TBCP group were identified to be at lower risk of developing BRD and thus not receiving BRD control therapy at processing. However, at 60 d on feed and at closeout, the PC displayed a significant reduction in BRD morbidity to both the NC and the TBCP system. Additionally, at closeout, the PC reduced overall mortality, BRD mortality, overall fallouts, and BRD fallouts compared to the NC. These findings subsequently led to differences in total kilograms (live and carcass weight) at closeout compared to the NC. The PC was not statistically different than the TBCP technology among those outcomes (possibly due to not including blocks 1 and 2 in the BRD control analyses), but large numerical differences were observed (**Table 5**) suggesting that findings observed in a prior study (Nickell et al., 2021) did not translate to the current study. These results are not necessarily surprising as prior literature reviews have not observed viable alternatives to BRD control antimicrobial therapy (Ribble et al., 2010; Cusack, 2024). Although antimicrobial use in food animal production may be scrutinized by the public, we must be mindful of consequences in removing a sound practice without a viable alternative. As these data indicate, eliminating BRD control antimicrobial application without a valid solution (even in a targeted manner) may create immediate economic and animal welfare concerns.

Prior research has repeatedly displayed poor diagnostic accuracy when BRD is detected by human observation and confirmed by a rectal temperature threshold (Wittum et al., 1996; Bryant et al., 1999; Gardner et al., 1999; Reinhardt et al., 2009; White and Renter, 2009; Rezac et al., 2014; Timsit et al., 2016). Therefore, advancements in disease detection technologies may improve upon the fallacies of human observation. In this study, compared to the traditional PR detection method, the ADD technology displayed significant improvements in health (both at 60 DOF and at closeout; **Tables 4 and 5**, respectively), performance (**Table 6**), pounds of HCW per head placed (**Table 7**), and ultimately economic outcomes. The economic advantage of the ADD system was observed to be $29.50/heifer compared to the PR group. This economic advantage was driven predominately by an increase in total HCW sold per pen (**Table 7**). However, unlike weight gain that is enhanced by steroid implants and beta-agonist products to the individual animal, the cumulative weight advantage observed in the ADD group in this study was attributed to a significant decrease in animals falling out of production prior to harvest compared to the PR group (**Table 5**). These results indicate the ADD system provided a greater economic return compared to the traditional modality of disease detection (i.e., pen riding) among the sample population enrolled in this study. Nonetheless, ongoing research is warranted to assess the repeatability of the technology and the robustness of its economic viability across populations experiencing different levels of disease pressure (Wolfger et al., 2015a).

Prior research describing the timing of BRD onset in the feedlot estimates that 75% of cases occur within the first 40 to 55 d on feed (Thompson et al., 2006; Babcock et al., 2009; Vogel et al., 2015). In the current study, similar dynamics were observed as the average day of BRD treatment occurred at 16 and 20 d on feed for the ADD and PR group, respectively (Table 5) while 98% of the BRD cases among both groups were diagnosed and treated by the time of reimplant (Day 80; data not shown). As described previously, the timepoint of reimplant provides a natural touchpoint where ADD tags may be removed and subsequently reused within a future population. The economic model in this study then assumed that method of application. However, to estimate retention duration and tag tolerance, tags were left in until harvest for the purposes of this study. Therefore, a post hoc economic analysis was performed accounting for the extended duration of use (224 d) assuming 1.5 uses/year (rather than 3) which increased the per head tag costs from $9.28 to $14.27. Under this scenario, the net economic advantage of monitoring cattle by the ADD system was reduced from $29.50/head to $24.51/head (*P* = 0.11) compared to the PR group.

Prior studies involving feeding behavior (Wolfger et al., 2015b), automated temperature measurement (Timsit et al., 2011a, 2011b; Theurer et al., 2013; McCorkell et al., 2014; Flattot et al., 2021), and generalized activity tracking (White et al., 2015, 2017, 2023) have displayed the ability to detect affected cattle several days prior to human observation of clinical signs. Likewise, in the closeout results for the current study, the ADD system detected calves requiring BRD therapy 4 d earlier (on average) compared to the PR. However, the results of the current study suggest the fate of an affected animal’s final disposition may be multifactorial rather than hinging solely on the timeliness of detection. In the current study, the ADD system displayed not only a shortened timeframe to first BRD treatment, but also a significant reduction in BRD pen-deads (i.e., a calf found dead without history of BRD diagnosis and treatment) compared to the PR. Given the challenges in defining a true gold standard for the BRD diagnosis, reducing a specific segment of BRD mortality suggests the ADD system has the potential to improve diagnostic sensitivity compared to PR methods. Concerns with diagnostic sensitivity of traditional pen-riding practices are well documented. Previous research has estimated the diagnostic sensitivity of pen-riding practices to be 61.8% (White and Renter, 2009). Likewise, subsequent research reported a much more conservative (yet variable) estimate of 27% (Timsit et al., 2016). Additionally, several prior observational studies have reported a proportion of the study population with lung pathology at harvest without history of BRD treatment (Wittum et al., 1996; Gardner et al., 1999; Thompson et al., 2006). Lastly, if additional BRD therapy was required, calves monitored by the ADD technology also were retreated faster (6 dearlier) than the PR calves (**Table 5**). In total, compared to cattle monitored by the PR, these cumulative findings suggest the observed efficacy of the ADD system may be multifactorial and associated with refinement of multiple clinical attributes that include an improvement in initial detection timeliness (i.e., time to first diagnosis and treatment), a potential improvement in diagnostic sensitivity (i.e., reducing the probability of false-negative outcomes), and (if needed) improved timeliness of retreatment.

In this study, BRD treatment success was comprised of three outcomes specifically defined as: 1) an animal that was diagnosed and treated for BRD but did not require retreatment, 2) was not removed for chronic BRD, and 3) did not die due to BRD. In the current study, no differences were observed between the disease detection treatments for both BRD retreatments and case-fatality at either the 60 dor closeout timepoints (**Tables 4 and 5**, respectively). Conversely, the risk of being culled (i.e., removals) due to chronic BRD was significantly reduced among cattle monitored by the ADD technology compared to those in the PR group. These observations were not unexpected. Prior research has observed no differences in BRD retreatment and mortality risk when comparing a different ADD system to conventional pen riding practices (White et al., 2015). Conversely, in a separate population, researchers observed a significant improvement in BRD retreatment and third treatment risk among cattle monitored by an ADD system compared to cattle monitored in a traditional manner (White B.J., 2017). Prior retrospective cohort studies have described BRD treatment failure (as defined above) as an outcome comprised of multiple factors manifesting both independently as well as modified across other parameters (Reinhardt et al., 2009; Babcock et al., 2010; Avra et al., 2017). Arrival body weight, quarter of arrival, BRD risk classification, and days on feed at first BRD treatment have all previously displayed a significant relationship with the risk of BRD treatment failure. Based on the findings of the current study as well as the outcomes of prior research, earlier and perhaps more accurate disease detection may not fully address the variation surrounding BRD retreatment and case-fatality risk. These observations may further affirm the need for an ongoing multi-factorial approach to BRD management that includes not only animal health and management practices at the feedlot but also ongoing preventative medicine efforts during the preweaning phase of production.

In the current study, the ADD system displayed a significant reduction in animals falling out of production prior to harvest compared to the PR (Table 5). However, several performance and carcass metrics such as final body weight and HCW tended (*P* > 0.05 - ≤ 0.10) to favor the PR (Tables 6 and 7, respectively). It is the authors’ interpretation that the remaining animals in the PR group (i.e. cattle remaining that did not die or had not been removed due to chronic BRD) represented a relatively healthy population leading to potential improvement in those respective performance and carcass metrics compared to the calves remaining in the ADD group. In contrast, the reduction in these performance metrics among calves in the ADD group may reflect a population who, potentially due to the ADD attributes discussed above, were healthy enough to finish production but who’s live and carcass performance was compromised. Nonetheless, despite the numerical differences in the above metrics, more total cattle monitored by the ADD system remained through harvest ultimately generating more final product (i.e., kg of beef) compared to calves monitored by the PR (Table 7). Ongoing research is necessary to determine if additional performance can be captured among calves monitored by the ADD system.

An interaction (or effect modification) can be defined as the combined effect on the respective outcome (i.e., response variable) of two (or more) treatments (i.e., independent variables) differing from the sum of the individual effects (Dohoo and Stryhn, 2003). In this study, no significant interactions were observed between BRD control and disease detection treatments among health outcomes at either timepoint. As discussed above, the positive control treatment within the BRD control factor and the ADD treatment within the disease detection factor displayed superior results when compared to their respective counterparts. In this study, one can conservatively presume the combination of this traditional BRD control program (i.e., administering tildipirosin to 100% of the population) and the disease detection method executed by the ADD technology resulted in additive value, rather than synergistic value, among calves in this sample population. Further research is warranted to investigate potential relationships between initial BRD control strategies and subsequent disease detection methods in the feedlot.

At the time of this writing, labor shortages are highly prevalent in multiple sectors of US agriculture (Zahniser et al, 2018). In feedlot production, personnel with experience differentiating normal from abnormal cattle have traditionally served as the method of disease detection. In addition, these detection skills must be applied across potentially thousands of animals and in various conditions. Concerns regarding the number of individuals willing to perform these tasks and the adequate skillset to sustain traditional outcomes have been posed (Enger et al, 2021). The current study suggests the ADD system can be implemented to improve disease detection efforts among populations like the one described herein. However, human labor is still required to act on the information and address issues beyond disease detection. Therefore, it is not the authors’ intent to suggest the ADD system is intended to replace people and eliminate employment. Rather, the ADD system is intended to be a tool for feedlot personnel to optimize animal outcomes and their own effectiveness. Nonetheless, future research is warranted to quantify the labor dynamics of the ADD system compared to traditional pen-riding practices.

Bovine viral diarrhea virus and its relationship with BRD in the feedlot is well described in the veterinary literature (Campbell, 2004; Loneragan et al., 2005; Booker et al., 2008; Larson, 2015). As stated above, four calves were identified as being PI-BVDV and were removed within 24 to 48 h after diagnosis. However, it is possible that transient BVDV infection (i.e., among non-PI calves in the original truck load and the subsequent pen mates) had an impact on the BRD outcomes within this study (Loneragan et al., 2005). With only four PI calves, even distribution across treatments and pens was not possible and the study was not designed around BVDV management. In addition, further diagnostic testing was not performed on morbid or dead cattle (beyond gross necropsy) in this study.

A limitation of this study reflects the differences in the BRD case definition between the ADD and PR treatment groups. Specifically, unlike the ADD system, the PR BRD case definition included a minimal rectal temperature criterion before treatment was permitted among calves allocated to the PR group. As stated above, a gold standard diagnosis for BRD does not exist. However, prior BRD antimicrobial development efforts and market support research have implemented a case definition similar to (or exactly like) the one used in the current study within the PR group. Therefore, much of our knowledge surrounding BRD therapy (i.e., both BRD control and the application of antimicrobial treatment) involves a case definition of the disease that constitutes our baseline standard of comparison for antimicrobial efficacy. That said, this likely explains why the ADD system displayed a reduction (*P* ≤ 0.05) in rectal temperature at the first BRD treatment compared to the PR group at both the 60-day and closeout time points (**Tables 4 and 5**, respectively). Future research comparing the ADD system to different permutations of a PR BRD case definition may be warranted.

## CONCLUSION

The current study provides further confirmation that the traditional BRD control program was superior to a negative control, but also was superior to a technology designed as a targeted BRD control program. Additionally, the ADD system displayed superiority in multiple health and performance metrics compared to the traditional PR method, ultimately leading to improved economic outcomes. No statistical interactions between BRD control strategies or disease detection modalities were observed in this study suggesting a traditional BRD control program and a novel ADD technology provided additive value within this study population. Stakeholders within the beef production industry are encouraged to retain proven tools while considering new opportunities designed to bring added value compared to traditional modalities.
